# Phosphorylation, Signaling, and Cancer: Targets and Targeting

**DOI:** 10.1155/2015/601543

**Published:** 2015-10-21

**Authors:** Sandra Marmiroli, Doriano Fabbro, Yoshihiko Miyata, Mariaelena Pierobon, Maria Ruzzene

**Affiliations:** ^1^Cellular Signaling Unit, Department of Surgery, Medicine, Dentistry and Morphology, University of Modena and Reggio Emilia, Largo del Pozzo, 71-41100 Modena, Italy; ^2^Piqur Therapeutics, Hochbergerstrasse 60C, 4057 Basel, Switzerland; ^3^Department of Cell and Developmental Biology, Kyoto University, Kitashirakawa, Oiwake-cho, Sakyo-ku, Kyoto 606-8502, Japan; ^4^Center for Applied Proteomics and Molecular Medicine, George Mason University, 10920 George Mason Circle, Manassas, VA 20110, USA; ^5^Department of Biomedical Sciences, University of Padova, Via U. Bassi 58/b, 35131 Padova, Italy

After 60 years from the first report of an enzymatic phosphorylation of proteins, protein kinases are well-established key signaling molecules that impact all major biological processes (reviewed in [1, 2]). Protein and lipid kinases fulfill essential roles in many signaling pathways that regulate normal cell functions [1–5]. Deregulation of kinase activities leads to a variety of pathologies ranging from cancer to inflammatory diseases, diabetes, infectious diseases, cardiovascular disorders, and cell growth and survival [1, 2, 5–11]. A much larger proportion of additional kinases are present in parasites and bacterial, fungal, and viral genomes that are susceptible to exploitation as drug targets [12]. Since many human diseases result from overactivation of protein and lipid kinases due to mutations and/or overexpression, this enzyme class represents an important target for the pharmaceutical industry [6]. Approximately one-third of all protein targets under investigation in the pharmaceutical industry are protein or lipid kinases and to date 33 small molecular weight kinase inhibitors (SMWKIs) and a handful of therapeutic antibodies have been approved for various indications mainly in oncology and many more in various stages of clinical and preclinical development [5]. Kinase inhibitor drugs, which are in clinical trials, target all stages of signal transduction from the receptor protein tyrosine kinases that initiate intracellular signaling, through second-messenger dependent lipid and protein kinases and protein kinases that regulate the cell cycle [10, 13]. While treating chronic phase CML (an almost monogenic disease) with imatinib has been very successful, the treatment of more advanced cancers with kinase inhibitors has proven more difficult due to the heterogeneity of these cancer types as well as due to kinase inhibitor resistance resulting from selection for mutant alleles and/or upregulation of alternative signaling pathways [5, 10].

In addition, powerful broad-scale protein pathway activation mapping and phosphoprofiling of protein kinases and of their specific substrates are contributing to driving the new concept of precision medicine [14–16]. Notable advances have been made in the rational design of small-molecule inhibitors that target unique kinase conformational forms and binding sites and have specific kinase selectivity profiles [5, 17–19].

This special issue provides a selection of original articles focused on kinase targets and therapeutic approaches based on kinase targeting in different cell systems and pathological conditions.

Resistance is a major drawback of conventional chemotherapy. The understanding of the mechanisms by which some cancer cells are resistant to ATRA (all-trans-retinoic acid) is crucial in anticancer drug development and requires detailed knowledge of the signaling pathways affected by ATRA. In the paper by R. S. Q. Barceinas et al., the authors show that, in lung cancer cells, ERK pathway activation is involved in the transcription-independent effects of ATRA. They also found that ERK targeting restores the beneficial effects of ATRA, suggesting that combined treatments with ATRA and ERK inhibitors could represent a promising strategy in lung cancer therapy.

Targeting of pathways aberrantly activated in cancer is a very powerful approach that has provided promising results, with several drugs already in clinical trials for a number of diseases. The work by M. Civallero et al. provides an excellent example of preclinical testing and comparison of the cytotoxicity of two different kinase inhibitors, namely, the pan-PI3K inhibitor BKM120 and the dual PI3K/mTOR inhibitor BEZ235, on mantle, follicular, and T-cell lymphomas, in which the PI3K pathway is known to be hyperactive. They demonstrated that BKM120 and BEZ235 show high effectiveness against lymphoma cells* in vitro* both as monotherapy and in combination. Because the outcome of lymphoma patients is still very poor, in spite of the great advances following the introduction of monoclonal antibodies based therapy of lymphoma, the results of this work open a new therapeutic window for these malignancies.

Along the same lines, the original work by C. Frasson et al. shows the pivotal role played by the PI3K/Akt pathway in promoting survival of primary medulloblastoma cells, the most frequent primitive neuroectodermal tumor in children, with a dismal prognosis in more than one-third of patients. In particular, the authors demonstrate that specific targeting PI3K impairs medulloblastoma primary cells growth and survival through the activation of the mitochondrial cell death program. Remarkably, PI3K signaling suppression seems to selectively hit the cancer stem cell population, sparing the most differentiated cellular counterpart. Since cancer stem cells have been reported to be one of the driving forces of tumor progression and resistance to therapy, these results may guide the development of new combinatorial therapeutic strategies aimed at impairing the survival machinery of both cancer stem cells and tumor bulk cells.

The acidophilic kinase CK2 has been shown to play prominent roles in carcinogenesis. In the paper by C. Girardi et al., the authors analyze two inhibitors of CK2, namely, TDB and CX-4945. In particular, they compare their efficacy in terms of persistence of inhibitory effects after their removal. They find a superiority of TDB compared to CX-4945, and they conclude that this property is an added value to be considered when planning new therapies based on CK2 targeting.

Inhibition of CK2 is also the focus of the paper by G. Cozza et al., where the authors analyze the properties of quinalizarin, extending its selectivity profile to 140 protein kinases. Consistent with* in silico* and* in vitro* analyses, they conclude that quinalizarin not only is one of the most selective inhibitors of CK2 but is also able to discriminate between the isolated CK2 catalytic subunit and CK2 holoenzyme.

A different aspect of the Akt kinase functions has been explored by G. Vallejo-Flores and coauthors. Based on the observation that* H. pylori* infection is the most important environmental risk to developing gastric cancer, mainly, through its virulence factor CagA, they evaluated the capacity of* H. pylori* CagA positive and negative strains to alter nontransformed glandular acini formation. They found that the Akt and BIM signaling pathway is activated in CagA positive strains and might thus contribute to its oncogenic activity through evasion of anoikis.

Overall, this special issue highlights the contribution of targeting signaling pathways in tumorigenesis and describes which compounds are more promising in the future for chemoprevention and anticancer therapy on the basis of their ability to inhibit specific signaling targets.

*Sandra Marmiroli* *Doriano Fabbro* *Yoshihiko Miyata* *Mariaelena Pierobon* *Maria Ruzzene*
